# Exploring the consequences of social defeat stress and intermittent ethanol drinking on dopamine dynamics in the rat nucleus accumbens

**DOI:** 10.1038/s41598-017-18706-y

**Published:** 2018-01-10

**Authors:** Alex L. Deal, Joanne K. Konstantopoulos, Jeff L. Weiner, Evgeny A. Budygin

**Affiliations:** 1 Department of Neurobiology and Anatomy, Wake Forest School of Medicine, Winston-Salem, NC USA; 2 Department of Physiology and Pharmacology, Wake Forest School of Medicine, Winston-Salem, NC USA; 3 Institute of Translational Biomedicine, St. Petersburg State University, St. Petersburg, Russia

## Abstract

The current study aimed to explore how presynaptic dopamine (DA) function is altered following brief stress episodes and chronic ethanol self-administration and whether these neuroadaptations modify the acute effects of ethanol on DA dynamics. We used fast-scan cyclic voltammetry to evaluate changes in DA release and uptake parameters in rat nucleus accumbens brain slices by analyzing DA transients evoked through single pulse electrical stimulation. Adult male rats were divided into four groups: ethanol-naïve or ethanol drinking (six week intermittent two-bottle choice) and stressed (mild social defeat) or nonstressed. Results revealed that the mild stress significantly increased DA release and uptake in ethanol-naïve subjects, compared to nonstressed controls. Chronic ethanol self-administration increased the DA uptake rate and occluded the effects of stress on DA release dynamics. Bath-applied ethanol decreased stimulated DA efflux in a concentration-dependent manner in all groups; however, the magnitude of this effect was blunted by either stress or chronic ethanol, or by a combination of both procedures. Together, these findings suggest that stress and ethanol drinking may promote similar adaptive changes in accumbal presynaptic DA release measures and that these changes may contribute to the escalation in ethanol intake that occurs during the development of alcohol use disorder.

## Introduction

It is well known that a wide range of stressful events can effectively trigger alcohol drinking behaviors in humans^1–3^. This phenomenon can be reproduced in several animal models of alcohol use disorder (AUD). For example, studies in primates revealed that stress via social separation significantly increased ethanol consumption even in monkeys not particularly vulnerable to fear-related behaviors^4^. Similar stress-related effects on ethanol drinking behavior can also be observed in rodent models of AUD. Thus, mice and rats exposed to social defeat episodes or socially isolated during adolescence show a persistent escalation in two-bottle choice drinking paradigms^5–10^.

In fact, both ethanol consumption and stress exposure induce overlapping neuroadaptations in several brain circuitries, including the mesolimbic dopaminergic pathway^11–15^. For example, *ex vivo* neurochemical studies have reported that both contingent and non-contingent chronic ethanol exposure^16–19^ as well as early-life social isolation stress^9,20^ trigger increases in dopamine (DA) reuptake rate in striatal subregions, including the nucleus accumbens. This outcome may result (at least in part) in a functional deficit in tonic DA release. Indeed, both stress and chronic ethanol exposure promote decreases in extracellular DA levels measured by microdialysis^15,21^. Based on these and other findings, it has been suggested that these conditions can induce a hypodopaminergic state that may alter the perception of rewarding and aversive effects of ethanol. Moreover, such neuroadaptations may contribute to the escalation in ethanol drinking behavior that drives AUD. However, relatively few studies have examined the effects of voluntary ethanol drinking on DA signaling dynamics or have examined possible interactions between the effects of stress and chronic ethanol intake on mesolimbic DA transmission, particularly in standard outbred animals. Therefore, the primary aim of the current study was to explore the effects of a relatively mild social defeat stress paradigm and an intermittent home-cage drinking procedure, which promotes moderate levels of ethanol intake, on DA dynamics in the nucleus accumbens. This study also sought to determine if a history of ethanol self-administration influenced any DA neuroadaptations induced by social defeat stress or if either procedure altered the acute effects of ethanol on accumbal DA release. These questions were addressed using fast-scan cyclic voltammetry (FSCV) coupled with single pulse electrical stimulation to evaluate changes in DA release and uptake parameters in the rat nucleus accumbens. These measures were performed on brain slices obtained from rats that were ethanol-naïve or chronically exposed to an intermittent home-cage ethanol drinking regimen and subsequently exposed or not exposed to stress (social defeat).

## Results

### Ethanol self-administration abolishes stress-induced increase in evoked DA release in the nucleus accumbens

**Figure 1:**
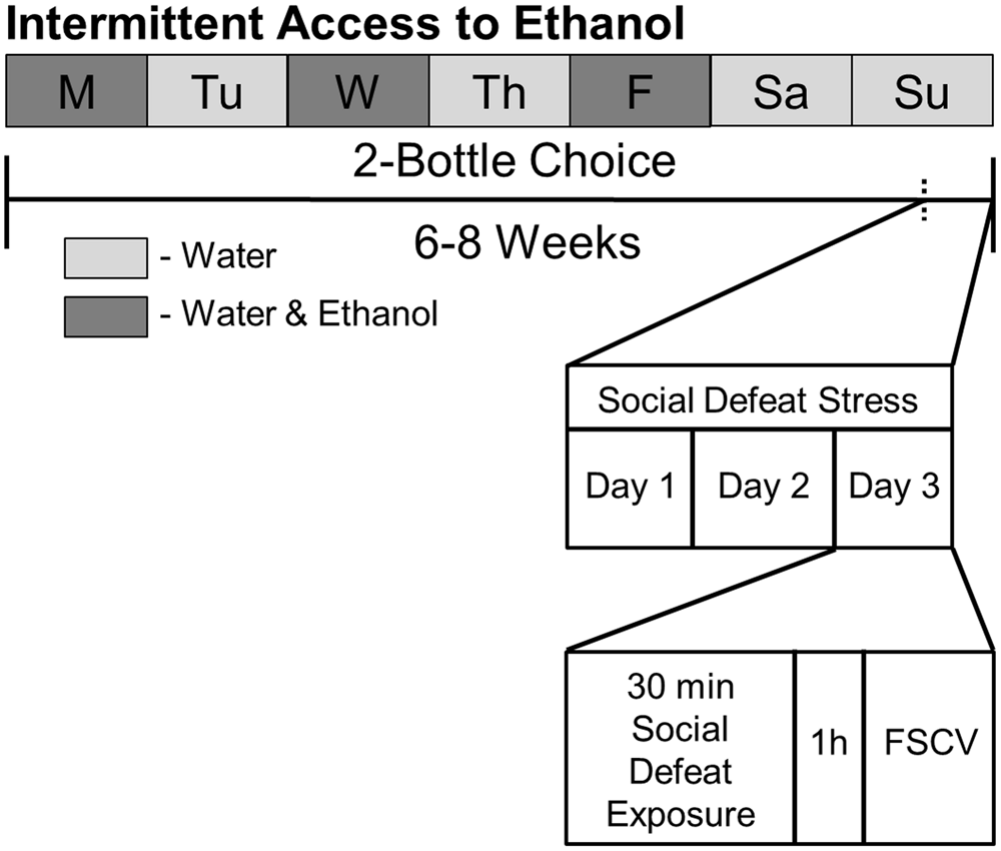
Schematic representation of performed experiments. Subjects were divided into 4 groups: Naïve, ethanol-exposed, stress-exposed, and exposed to both ethanol and stress rats. Briefly, ethanol-exposed subjects had access to water and ethanol on Monday, Wednesday, and Friday (dark boxes) and only water on other days (light boxes). After a minimum of 6 weeks, stress-exposed subjects underwent 3 days of social defeat stress exposure. One hour after the third social defeat stress session, subjects were taken for *in vitro* fast-scan cyclic voltammetry (FSCV).

**Figure 2:**
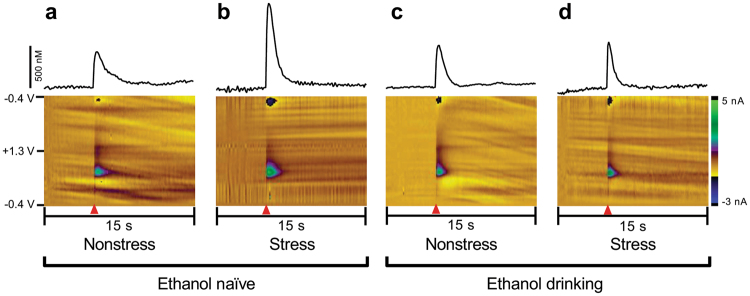
Representative traces of electrically-evoked accumbal DA release measured in brain slices from naïve (**a**), stressed (**b**), ethanol drinking nonstressed (**c**) or stressed (**d**) rats (upper panel). These signals had an oxidation peak at +0.6 V and reduction peak at −0.2 V versus an Ag/AgCl reference, identifying the released species as DA. Representative color plots topographically depict the voltammetric data, with time on the *x*-axis, applied scan potential on the *y*-axis, and background-subtracted faradaic current shown on the *z*-axis in pseudo-color (lower panel).  = electrical stimulus onset.

**Figure 3:**
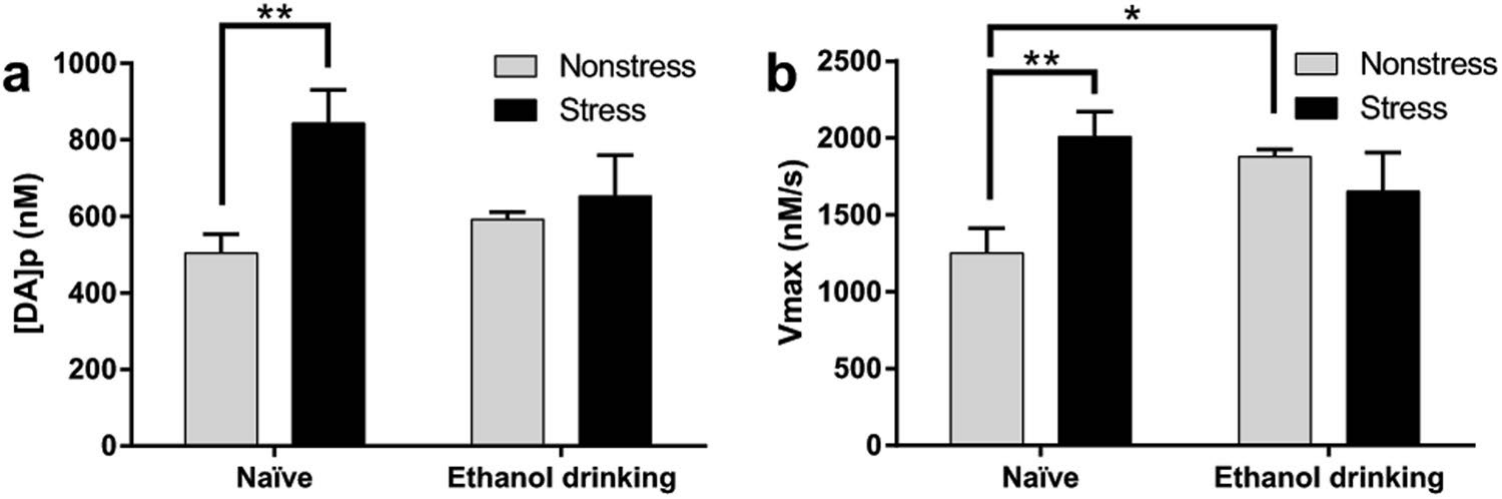
Prior ethanol drinking alters effects of social-defeat stress on DA dynamics in rat nucleus accumbens. Stressed subjects showed significantly higher DA release per stimulus pulse compared to nonstress control (A, left panel). Prior ethanol exposure completely blunted this effect (A, right panel). Both stress (B, left panel) and ethanol exposure (B, right panel) significantly increased accumbal DA uptake, measured as the *V*_max_. However, there was no significant difference between subjects exposed to defeat stress episodes and ethanol (B, left panel). Data presented as mean ± SEM and analyzed by a two-way ANOVA. A single slice was taken from each subject in every experimental group for FSCV recordings. The number of subjects for naïve nonstressed: n = 10; for naïve stressed: n = 9; for ethanol drinking and nonstressed: n = 5; for ethanol drinking and stressed: n = 5. *p < 0.5; **p < 0.01.

Figure 1 schematically represents the experimental design of the current experiments for ethanol-drinking rats (ethanol-naïve subjects underwent the same design with access to water only). Electrically-evoked DA release data, obtained with *ex vivo* FSCV, were analyzed for naïve (EN) and ethanol drinking (EE) rats that were either exposed to social-defeat stress episodes or served as nonstressed control animals (Figs 2 and 3a). The data were proved to be normally distributed following a Shapiro-Wilk test for normality (p > 0.05). A two-way ANOVA analyzing the differences in concentration of DA released per stimulus pulse showed a main effect of stress condition (F(1,25) = 6.562, *p* = 0.0168) but not ethanol experience (F(1,25) = 0.430, *p* = 0.518) and a trend towards a significant interaction (F(1,25) = 3.174, *p* = 0.0870). Tukey’s multiple comparisons test revealed that this parameter was significantly greater in ethanol-naïve subjects exposed to social defeat stress compared to ethanol-naïve subjects not exposed to stress (843.4 ± 86.6 vs 504.4 ± 49.4 nM; *p* = 0.0056; Fig. 3a). In marked contrast, social defeat stress episodes did not change DA release in animals with a prior history of intermittent ethanol drinking (592.3 ± 19.9 vs 653.2 ± 107.2 nM; *p* > 0.05; Figs 2 and 3a). There was also no difference in the magnitude of DA release between the nonstressed ethanol-naïve and ethanol-drinking cohorts (504.4 ± 49.4 vs 592.3 ± 19.9 nM; *p* > 0.05; Figs 2 and 3a). Therefore, prior ethanol drinking did not alter electrically-evoked DA release in nonstressed control subjects but did prevent the increase in evoked DA release promoted by social defeat stress in rat nucleus accumbens.

### Social defeat stress and ethanol self-administration accelerate DA uptake in the nucleus accumbens

In parallel with the analysis of the magnitude of DA release, the DA uptake rate, expressed as *V*_max_, was analyzed and compared between groups to determine the effects of social stress and ethanol self-administration on this parameter (Fig. 3b). The data were shown to be normally distributed by passing a Shapiro-Wilk test for normality (p > 0.05). A two-way ANOVA found a significant interaction (F(1,27) = 9.084; *p* = 0.0056) but no main effect of stress condition (F(1,27) = 2.673, *p* = 0.114) or ethanol experience (F(1,27) = 0.703, *p* = 0.409). As observed with DA release, Tukey’s multiple comparisons test showed significantly higher uptake rates in stressed ethanol-naïve subjects relative to their nonstressed controls (2008 ± 164 vs 1251 ± 162 nM/s; *p* = 0.0058; Fig. 3b). Interestingly, a history of ethanol drinking also significantly increased the rate of DA uptake (2008 ± 164 vs 1878 ± 49 nM/s; *p* = 0.0323; Fig. 3b) and blocked any further stress-associated enhancement of this parameter in the nucleus accumbens (1878 ± 49 vs 1653 ± 253 nM/s; *p* > 0.05; Fig. 3b).

### Stress and ethanol self-administration reduce effects of an acute ethanol challenge on electrically-evoked DA efflux

**Figure 4:**
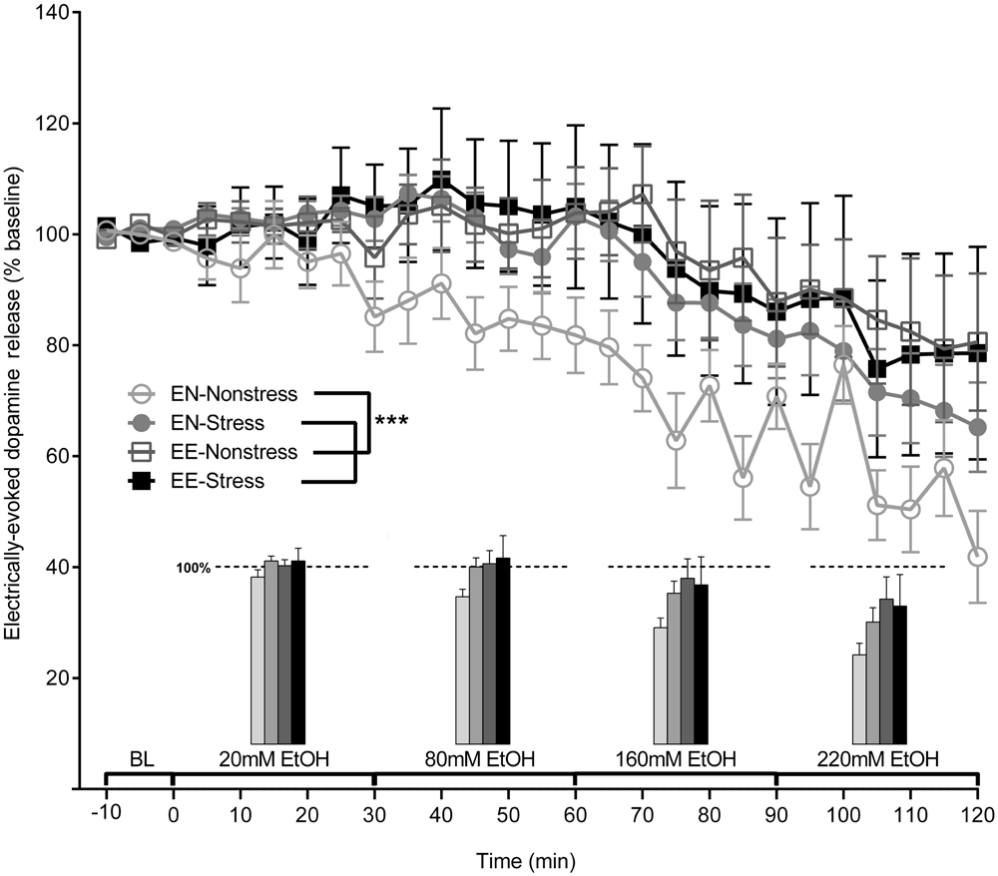
Prior ethanol exposure and social-defeat stress significantly attenuate the effects of acutely applied ethanol on electrically-evoked DA release in rat nucleus accumbens. The inserted bars above each ethanol concentration along the x-axis represent the averaged DA efflux observed during the final 20 min of each concentration as a percent of pre-ethanol values (dashed line = 100% of baseline). All data are presented as mean ± SEM and analyzed by a Friedman test. A single slice per rat was used for FSCV in every experimental group. The number of subjects for naïve nonstressed: n = 7; for naïve stressed: n = 10; for ethanol drinking and nonstressed: n = 6; for ethanol drinking and stressed: n = 5. ***p < 0.001.

The effects of acute ethanol on evoked accumbal DA release were compared across all experimental conditions. These DA release changes were plotted as a percent of the baseline signal collected prior to application of the first ethanol concentration (Fig. 4). Due to unequal variances between groups, a nonparametric analysis was used to evaluate these data. Using the Friedman test revealed a significant effect of condition (χ^2^(3) = 45.04, *p* < 0.0001). Furthermore, linear regression analysis of DA release changes during acute ethanol application found that all slopes for all groups were significantly different from 0 (*p* < 0.0001) and negative. Correlation coefficients (r^2^) were 0.856 (EN-Nonstress), 0.846 (EN-Stress), 0.664 (EE-Nonstress), and 0.685 (EE-Stress). Dunn’s multiple comparisons test found that the ethanol-naïve, nonstress group was significantly different than all other groups regarding the acute effect of ethanol on DA release (*p* < 0.001). However, no difference was found between EN-Stress, EE-Nonstress and EE-Stress groups (*p* > 0.5).

### Stress and ethanol self-administration do not modify evoked DA release recovery following an acute ethanol challenge

**Figure 5:**
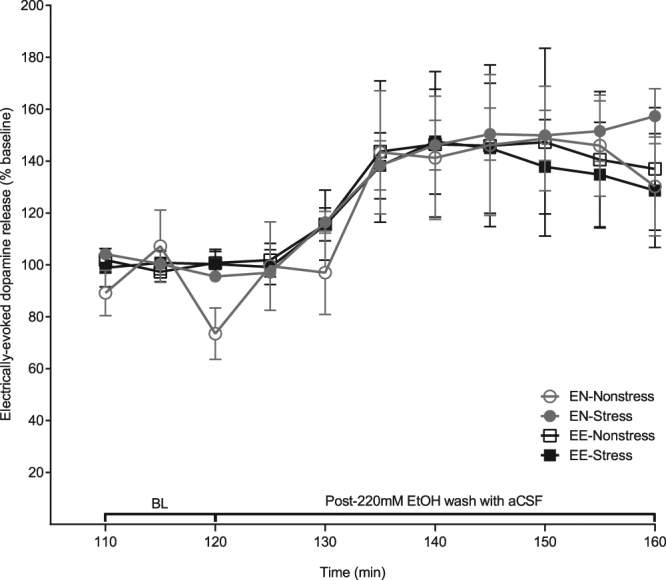
Stress and ethanol exposure do not affect electrically-evoked DA signal recovery during aCSF wash following ethanol application. After the final ethanol concentration (220 mM), the brain slices were perfused with aCSF containing no ethanol. There was no significant difference in the recovery of electrically-stimulated DA efflux between any groups after replacing the ethanol solution with aCSF. Data are presented as mean ± SEM and analyzed by a Friedman test. The number of subjects (a single slice per rat) for naïve nonstressed: n = 7; for naïve stressed: n = 10; for ethanol drinking and nonstressed: n = 6; for ethanol drinking and stressed: n = 5.

After the final ethanol concentration was perfused, we analyzed the recovery of the DA signal by washing aCSF over the slice (Fig. 5). The amplitudes of DA release from the final 3 time points of ethanol application at the 220 mM concentration were averaged to calculate a new baseline (100%). This approach allowed us to more accurately to compare the recovery of evoked DA release following acute ethanol exposure across all experimental conditions. Due to unequal variances between groups, a nonparametric test was used to analyze these results. According to the Friedman test, there was no significant difference between conditions (χ^2^(3) = 4.96, *p* > 0.05) during the recovery period. Therefore, neither defeat stress nor ethanol drinking experience or their combination resulted in any changes in DA signal recovery from the acute effect of ethanol applied locally in the nucleus accumbens.

## Discussion

The most fascinating finding of this study was that a relatively mild stress exposure (three social defeat episodes without any physical interaction) significantly altered evoked DA release and consequent uptake in accumbal brain slices. Moreover, we also found that a history of moderate ethanol self-administration through the two-bottle choice paradigm significantly increased the rate of DA uptake and that this drinking experience blunted the effects of social defeat stress on accumbal DA dynamics. Finally, we demonstrated that stress and ethanol self-administration decreased the acute effects of ethanol on evoked DA efflux in the nucleus accumbens and that ethanol drinking occluded additional effects of stress on this measure. No group differences were observed in the time course of the recovery of the DA response following acute ethanol exposure.

This study extends our earlier report that social defeat stress can increase the frequency of burst activity in the ventral tegmental area (VTA) and therefore increase phasic DA release in the nucleus accumbens of freely moving rats^22^. It is important to highlight that these changes remained after the confrontation terminated and the defeated animal was returned to its home cage^22^. Here we demonstrated that the consequences of this stressor on DA dynamics can also be detected in an *ex vivo* preparation, where DA efflux is triggered by electrical stimulation of terminals which are disconnected from the DA cell bodies. Therefore, relatively brief stress episodes can result in significant alterations in presynaptic DA activity. These results are in good agreement with previous *ex vivo* findings demonstrating enhanced evoked DA efflux and reuptake rate following adolescent social isolation, a well-established model of chronic early-life stress^9,20,23^. Studies using the social isolation model revealed that the increased DA uptake was accompanied by an increase in expression of the DA transporter. Together, these data demonstrate that different stressful events may promote analogous maladaptive changes in presynaptic DA dynamics.

Previous findings revealed a reduction in dynorphin peptide and dynorphin mRNA levels following social isolation and social defeat stress, respectively^9,24^. Importantly, activation of kappa opiod receptors (KORs), which are found on DA terminals, suppresses DA release in the nucleus accumbens^9,25,26^. Consequently, decreased levels of endogenous agonist could result in reduced inhibition of DA release through the activation of KORs. In addition to this possible mechanism, accumbal DA release can be increased via co-activation of corticotropin-releasing factor (CRF) 1 and CRF2 receptors^27^.

Dynorphin-DA interactions may potentially explain why prior ethanol exposure completely prevented the stress-induced DA increases. There is evidence for enhanced levels of dynorphin in rat brain regions, including the nucleus accumbens, following repeated ethanol administration^28,29^. Moreover, a recent study demonstrated that chronic ethanol exposure increased KOR responsiveness to agonist^30,31^. The combination of these changes might result in suppression of DA release, since KOR activation has clear inhibitory actions on accumbal DA release^26,30^. In fact, a reduction in electrically-evoked DA release following chronic ethanol vapor exposure was observed in mouse^19,30^, but not rat brain slices^17^. However, electrically-evoked DA release measured in the nucleus accumbens *in vivo* was not different between ethanol-naïve rats and an adolescent binge alcohol group^32^ and intermittent ethanol drinking did not alter accumbal DA efflux in the current study. The difference between species combined with the model of ethanol exposure and the time point when measurements were performed may account for these discrepancies. Perhaps, this effect of chronic ethanol exposure through two-bottle choice on electrically-evoked DA can be blunted in rats to a greater extent than in mice. For example, reduced accumbal D_2_ autoreceptor regulation in rats^33–35^ could obstruct this effect. Alternatively, chronic ethanol-induced supersensitivity of D_2_ autoreceptors combined with the increased reuptake rate observed in mouse nucleus accumbens^19,30^, but not adaptations in the KOR system, can be preferentially responsible for the DA decrease following chronic ethanol^19,30^.

This study investigated the influence of stress and chronic ethanol exposure through the two-bottle choice procedure on the acute effects of ethanol on DA release in male rats. Therefore, future studies using females are necessary to explore a possible sex difference in stress and alcohol interaction, since women are more susceptible to AUD than men. It should also be noted that we focused on the effects of relatively high ethanol concentrations, as *in vitro* FSCV studies can only detect the inhibitory effects of ethanol on DA release dynamics^16,36–39^, since DA cell bodies are not present in these preparations. In contrast, lower doses of ethanol have a well-characterized excitatory effect on DA transmission when administered *in vivo*. This occurs through an increase in neuronal firing rate in the ventral tegmental area^40,41^, thereby elevating DA release in the nucleus accumbens^42–45^. Therefore, any changes observed in the acute effects of ethanol in brain slices may preferentially reflect the development of tolerance to the inhibitory effect of ethanol on accumbal DA release. In fact, prior stress and chronic ethanol drinking, either alone or together, significantly reduced the acute effects of ethanol on electrically-evoked DA efflux in the rat nucleus accumbens in this study. These findings are consistent with other findings in the literature. For example, exposure to binge levels of ethanol during adolescence blunted ethanol inhibition of electrically-evoked DA release measured in the nucleus accumbens of adult rats *in vivo* ^32^. Similarly, a complete tolerance to the inhibitory effect of 150 mM ethanol on DA release was observed in caudate slices from monkeys which drank ethanol for 18 months^16^, although the ability of lower ethanol concentrations (80 and 120 mM) to decrease electrically-evoked DA release following seven months of the same drinking model was unchanged^46^. Together, these data suggest that the time required for the development of tolerance to the acute inhibitory effects of ethanol on accumbal DA release may vary in different species.

To our knowledge, this study is the first to report on the effects of stress exposure on ethanol inhibition of accumbal DA release. On the other hand, prior work has shown that social stress boosted the excitatory effects of ethanol on DA release in the rat nucleus accumbens^47^. The combination of enhanced excitation (at low to moderate ethanol doses) and blunted inhibition (at higher concentrations) observed in the present experiment may lead to decreased aversive and increased rewarding properties of ethanol. This shift could potentially accelerate ethanol drinking behavior and perhaps the development of earlier stage of addiction. In agreement with this suggestion, exposing mice and rats to social defeat episodes or social isolation during adolescence promotes increases in ethanol intake^5–10^. Together, these results suggest that social defeat stress and chronic ethanol drinking through the two-bottle choice paradigm trigger overlapping adaptations at DA terminals in the nucleus accumbens, which may promote maladaptive escalations in ethanol drinking behavior associated with alcohol addiction.

## Materials and Methods

### Animals and behavioral procedures

Single-housed male Long Evans rats (400–600 g; Envigo) were used in this study. Animals were kept on a 12-hr light/dark cycle (lights off at 6 pm). Food and water were available *ad libitum*. Subjects were assigned to either ethanol-naïve (EN) or ethanol-exposed (EE) and nonstress or stress groups. For the EE subjects, ethanol (20%) and water were available for 24 hrs through a two-bottle choice paradigm^48^ on each Monday, Wednesday, and Friday for 6–8 consecutive weeks. Water and ethanol bottles were alternated to account for side preference and weighed to quantify daily consumption. There was no difference in ethanol intake values measured for the entire drinking period between the nonstress and stress groups (3.1 ± 0.1 vs 3.5 ± 0.2 g/kg/day, respectively). The average ethanol intake for the week prior to voltammetric experiments for all subjects was 3.7 ± 0.3 g/kg/day. Notably, previous studies have revealed that this level of ethanol consumption generated pharmacologically relevant blood ethanol concentrations^49^. Ethanol was removed from the cages twenty-four hours before sacrificing the animals for neurochemical analyses.

Ethanol-naïve and ethanol-exposed subjects were stressed three times via a social defeat procedure on separate days. We used an open top, clear Plexiglas container (h = 18″; l = 54″; w = 24″) divided into two equal chambers with an opening (7″ × 7″) in the dividing wall connecting the two halves. “Intruder” stressed rats were confined within an immobile cylindrical metal cage (h = 8.25″; diameter = 10.75″) in the middle of one chamber while the “aggressor” rat was placed in the empty chamber. The social defeat stress bouts were conducted during the light phase around 9 a.m. and lasted for 30 min each. The “aggressor” rat was able to move freely around the testing apparatus, including up to the metal cage confining the “intruder” rat, but physical contact between the rats could not occur. The exposure of intruders was counterbalanced between different groups to minimize possible effects of habituation of the used aggressive residents. The same “aggressor” rat was used for all stress bouts for all “intruder” rats to control for variations in aggressive behaviors. Observations noted the “aggressor” would often stand up against the containment cylinder or place his front paws on top of the cylinder. Although direct physical contact between the “intruder” and “aggressor” was prevented^50^, these postures were sufficiently anxiogenic, as the “intruder” rat exhibited species-specific stress behaviors (e.g. hunched posture, decreased movement)^11,51^. The chambers were cleaned with a mild detergent and bleach solution between each stress procedure. The “intruder” rats (EN and EE stress groups) were further used for voltammetric experiments.

Animal handling and all procedures were conducted in accordance with the National Institutes of Health Guide for the Care and Use of Laboratory Animals. All protocols were approved by the Wake Forest University School of Medicine Institutional Animal Care and Use Committee.

### Fast-scan cyclic voltammetry

The “intruder” rats (EN, n = 10 and EE, n = 5 per group) were sacrificed 1 h following the third stress event. Nonstressed (EN, n = 7 and EE, n = 6) animals, matched by age and housing conditions, were taken at a similar time of day to be sacrificed, with nonstressed EN subjects serving as the control. Briefly, rats were anesthetized with isoflurane, decapitated and the brain was rapidly removed and immediately placed in ice-cold oxygenated artificial cerebrospinal fluid (aCSF): 126 mM NaCl, 2.5 mM KCl, 1.2 mM NaH_2_PO_4_ (monobasic), 2.4 mM CaCl_2_, 1.2 mM MgCl_2_, 0.4 mM L-ascorbic acid, 11 mM C_6_H_12_O_6_, 25 mM NaHCO_3_, and adjusted pH to 7.40. Coronal slices 400 µm thick containing the nucleus accumbens were obtained using a vibrating tissue slicer (Vibratome 1000 Plus, The Vibratome Company, St. Louis, MO, USA) and then placed in oxygenated aCSF at room temperature and allowed 30 min to equilibrate. The slices were taken and placed on a submersion recording chamber with room-temperature oxygenated aCSF flowing at a rate of 1 mL/min. Carbon fiber electrodes (diameter: 7 µm; Goodfellow Cambridge Ltd., Huntington, UK) were constructed as previously described^52^ and the exposed carbon fiber was trimmed to 90–145 µm with a scalpel under a microscope (Leica, Buffalo Grove, IL, USA). The carbon fiber electrode was connected to a voltammetric amplifier (UNC Electronics Design Facility, Chapel Hill, NC, USA) and placed into the nucleus accumbens core while a twisted bipolar stimulating electrode (Plastics One, Roanoke, VA, USA) was placed on the surface of the tissue ~200 µm from the recording electrode and connected to a voltage output box. DA release was evoked by a single, rectangular, electrical pulse (350 µA, 4 ms/phase, monophasic) applied every 5 min. Extracellular DA was recorded at the carbon fiber electrode every 100 ms for 15 s by applying a triangular waveform (−0.4 V to +1.3 V and back to −0.4 V vs Ag/AgCl, 400 V/s). DA was identified by observing background-subtracted cyclic voltammograms characterized by oxidation and reduction peaks occurring at ~+0.6 and ~−0.2 V, respectively (vs. Ag/AgCl reference). Following acquisition of a stable baseline DA signal (3 recordings within 10% variability), ethanol was washed over the slice at increasing concentrations (20 mM, 80 mM, 160 mM, and 220 mM ethanol in oxygenated aCSF) for 30 min each. Oxygenated aCSF was washed over the slice at the conclusion of the 220 mM exposure to measure DA signal recovery for 40 min. Data were digitized (National Instruments, Austin, TX, USA) and stored on a computer. The response of the carbon fiber microelectrode was calibrated in a flow injection analysis system after each experiment^53^. The calibrations were performed in triplicate using a known concentration (1 µM) of DA (Sigma Aldrich, St. Louis, MO, USA) that was dissolved in calibration buffer adjusted pH to 7.40 at room temperature. The voltammetric current was measured at the peak potential. The averaged calibration factor was 14 ± 3 nA per 1 µM of DA.

### FSCV kinetic analysis

DA release parameters (i.e., DA release per stimulus pulse ([DA]_p_) and uptake (*V*_max_)) of recordings prior to ethanol administration were calculated using kinetic modeling (LVIT software, UNC, Chapel Hill, NC, USA). These parameters were modeled assuming to follow Michaelis-Menten kinetics^17,54^. DA concentration changes with respect to time were estimated by equation (1):

1 $$d[DA]/dt=(f)[DA]p-(V\,\max \,/\{(Km/[DA])+1\})$$

where *f* is the stimulation frequency (Hz), [*DA*]_p_ is the concentration of DA released per stimulus pulse, and *V*_*max*_ and *K*_*m*_ are Michaelis-Menten rate constants for DA uptake. The baseline value of *K*_*m*_ was set to 0.16–0.20 µM, a value determined in rat brain synaptosomes^55^.

### Data analysis

Data were analyzed using GraphPad Prism (GraphPad Software version 6.05, San Diego, CA, USA). Two-way ANOVAs and Tukey’s multiple comparisons tests (where appropriate) were used to analyze DA release and uptake data. Due to unequal variances between groups, evoked DA efflux data were analyzed as a percent of baseline using nonparametric Friedman tests and Dunn’s multiple comparisons test (where appropriate). Data are presented as mean ± SEM and the criterion for significance was set at *p* < 0.05.

### Data availability

The datasets generated and analyzed during the current study are available from the corresponding author upon reasonable request.
